# Nanomolar Caffeic Acid Decreases Glucose Uptake and the Effects of High Glucose in Endothelial Cells

**DOI:** 10.1371/journal.pone.0142421

**Published:** 2015-11-06

**Authors:** Lucia Natarelli, Giulia Ranaldi, Guido Leoni, Marianna Roselli, Barbara Guantario, Raffaella Comitato, Roberto Ambra, Francesco Cimino, Antonio Speciale, Fabio Virgili, Raffaella Canali

**Affiliations:** 1 Council for Agricultural Research and Economics, Food and Nutrition Research Centre, Rome, Italy; 2 Department of Physics, Sapienza University of Rome, Rome, Italy; 3 Department of Drug Sciences and Health Products, University of Messina, Messina, Italy; University of Nebraska Medical Center, UNITED STATES

## Abstract

Epidemiological studies suggest that moderate and prolonged consumption of coffee is associated with a reduced risk of developing type 2 diabetes but the molecular mechanisms underlying this effect are not known. In this study, we report the effects of physiological concentrations of caffeic acid, easily achievable by normal dietary habits, in endothelial cells cultured in 25 mM of glucose (high glucose, HG). In HG, the presence of 10 nM caffeic acid was associated with a decrease of glucose uptake but not to changes of GLUT-1 membrane localization or mRNA levels. Moreover, caffeic acid countered HG-induced loss of barrier integrity, reducing actin rearrangement and FITC-dextran passage. The decreased flux of glucose associated to caffeic acid affected HG induced apoptosis by down-regulating the expression of initiator (caspase 8 and 9) and effector caspases (caspase 7 and 3) and by increasing the levels of phosphorylated Bcl-2. We also observed that caffeic acid in HG condition was associated to a reduction of p65 subunit nuclear levels with respect to HG alone. NF-κB activation has been shown to lead to apoptosis in HG treated cells and the analysis of the expression of a panel of about 90 genes related to NF-κB signaling pathway revealed that caffeic acid significantly influenced gene expression changes induced by HG. In conclusion, our results suggest that caffeic acid, decreasing the metabolic stress induced by HG, allows the activation of survival mechanisms mediated by a different modulation of NF-κB-related signaling pathways and to the activation of anti-apoptotic proteins.

## Introduction

Persistent hyperglycemia, a chronic increase of blood glucose levels, is one of the most evident features of type 2 diabetes (T2D). At cellular level, high glucose (HG) significantly affects the metabolic homeostasis and is associated to a progressive failure of several tissues and of the system response, including immune response and vascular function [1]. Endothelium is profoundly affected by HG as it is the first tissue exposed to changes of glucose levels. A chronic and prolonged exposure to elevated glucose levels has been recognized to cause endothelium dysfunction and correlated diseases, from blindness to atherosclerosis [2]. At molecular level, HG has been reported to impair cellular responses associated to increased permeability [3, 4] and to a generalized condition of vascular inflammation, eventually leading to cell death [5, 6]. The activations of protein kinase C (PKC), hexosamine flux [7] and NF-κB activation [8] have been reported as mainly effectors of HG in endothelial cells.

Together with wine and tea, coffee is one of the most widely consumed polyphenol and phenolic acid rich beverages in the world. Hydroxycinnamic acids are the major class of phenolic acids in coffee. Among them, chlorogenic acid that is eventually metabolized to caffeic acid (CA), *p*-coumaric acid and ferulic acids are the most represented ones. Health benefits of polyphenols have been widely claimed on the basis of the evident inverse relationship linking the consumption of polyphenols-rich food items with a wide spectrum of degenerative diseases, including cardio-vascular disease and cancer, and chronic inflammation [9, 10]. More specifically, several epidemiological studies reported that moderate and prolonged consumption of coffee is associated with a reduced risk T2D [11, 12]. However, in spite of the general agreement about the potential beneficial effects of coffee polyphenol consumption, only few *in vitro* studies considered their real bioavailability in humans and therefore no information are available about the effects of physiological concentrations of CA. This aspect is obviously fundamental for the understanding of the implications of dietary phenolic acids in human health and to clarify the mechanism by which they exert their protective role.

Available data clearly indicate that the plasma concentration of any molecule, either the parental one or metabolites, resulting from the consumption of 10–100 mg of a single compound after coffee consumption rarely exceeds 1 μM [13]. In particular, plasma conjugated and aglycone CA levels have been reported in the range of 300–500 nM and 10–116 nM respectively, after consumption of 200 ml of American coffee [14]. Irrespective of this evidence, the majority of the studies addressing the effects of phenolic acids on vascular cells consider much higher concentrations (most frequently from micromolar to millimolar) therefore introducing a strong and evident bias, possibly resulting in misleading results.

We have therefore tested the effects of nanomolar concentrations of CA, easily achievable by normal dietary habits, on HG-induced dysfunction of endothelial cells. Glucose concentration was set in order to mimic the circulating levels typical of T2D-associated hyperglycemia. The human vascular endothelial cell line Ea.hy926, derived from the fusion of lung adenocarcinoma cells A549 with primary human umbilical vein endothelial cells (HUVECs), was utilized to demonstrate that nM concentrations of CA significantly counter several dysfunctional effects of HG.

## Materials and Methods

### Cell culture and experimental design

The human stabilized endothelial cell line Ea.hy926 was kindly provided by Dr. C.J. Edgell (University of North Carolina, Chapel Hill, NC, USA) [15]. Cells were cultured in DMEM with 5.5 mM glucose (EuroClone, Italy) supplemented with 10% inactivated FCS, 2 mM L-Glutamine, 100 IU/ml penicillin, 0.1 mg/ml streptomycin and 2% HAT (Hypoxanthine, Amnopterin, Thymidine). Cells were maintained at 37°C in 5% CO_2_. Once sub-confluence was reached, cells were exposed to media containing either 5 mM D-glucose (control), or 25 mM D-glucose (HG). Caffeic acid was added at a concentration of 10 nM either to control (CA) or to HG (CA+HG) media. Incubation times are specified in the text. The concentration of CA was chosen on the basis of preliminary experiments in which three different concentrations (5,10 and 100 nM) of the phenolic acid were tested on HG dependent decrease of cell viability measured by Trypan Blue exclusion test; 10 nM corresponds to the lowest concentration of CA associated to cell protection against HG-induced cell mortality (S1 Fig).

### Glucose uptake assay

Sub-confluent cells were plated in a 12 well plate and treated as described above, for 30 minutes or 3, 6, 24 and 48 hours. At the end of the incubations cells were washed two times with PBS and then incubated with 500 μl PBS containing 100 μM 2-deoxy-D-glucose (dGlu) and 0.1 μCi/ml 2-deoxy-D- [C14]glucose (specific activity 57.8mCi/mmol) (Perkin Elmer, MA, USA). After 5 minutes of incubation at 37°C, cells were washed three times in ice cold PBS and solubilized in 0.4 ml of 1% SDS and an aliquot was used to measure the protein amount using the Bio-Rad DC^™^ Protein Assay (Bio-Rad, CA, USA). Cell lysate radioactivity was measured by scintillation counting using a Perkin Elmer counter. The rate of glucose was expressed in pmol dGlu/min/μg protein and shown in the figure as % of control at each time point.

### FITC-Dextran assay

In order to evaluate the effect of HG and CA on paracellular permeability, endothelial cells were seeded on transwell polyethylene terephthalate (PET) membrane filters (12-well format, BD FALCON^™^ Cell Culture Inserts, 12 mm diameter, pore size 0.4 μm) and cultured as described above for 24, 30, 48 and 72 hours. The passage of 40 kDa fluorescein isothiocyanate (FITC)-labeled dextran (FD40, BD Pharmingen^™^, Italy) from the apical to basal compartment of the filter was measured. FD40 (500 μg/ml) was added to the apical compartment 45 minutes before the end of the incubation. At the end of incubation, basal and apical media were collected and analyzed in a fluorescence detector (Infinite^®^ 200 PRO, TECAN, Switzerland) using 490 nm and 520 nm as excitation and emission wavelengths, respectively. FD40 values are expressed as percentage of FD40 basal/apical ratio [16].

### Immunofluorescence for F-actin organization

Cells were plated on 12-well format membrane filters and cultured as described above for 48 hours. Immunofluorescence experiments were performed by standard protocols on cells fixed in 2% paraformaldehyde (PFA) after permeabilization in 0.1% TRITON X-100. F-actin was stained with FITC-conjugated phalloidin (Sigma-Aldrich, MO, USA). For nuclear staining, 300 nM 4′,6-diamidino-2-phenylindole dihydrochloride (DAPI) (Sigma-Aldrich, Italy) was added directly to the mounting medium. Specimens were analyzed using an inverted laser-scanning confocal microscope equipped with a 40× oil-immersed objective (LSM 700; Carl Zeiss, Germany). Serial optical sections were processed with ZEN 2009 software (Carl Zeiss, Germany). [17]

### Cell number and proliferation

Cells were seeded in 6-well plates and treated for 24, 48 and 72 hours as described above. At the end of the incubation, cells were counted using a digital inverted-microscope (EVOS^®^ microscope, AMG, Advanced Microscopy Group, Washington, USA) equipped with an integrated computer and imaging software designed and programmed for real-life imaging. After images acquisition of different fields per well, cells were counted using 7x8 grid. Data are expressed as number of cells/well.

Proliferation of Ea.hy926 cells was assessed by measuring the incorporation of 5-bromo-2’-deoxyuridine (BrdU). Cells were plated in 96-well plate and pulse-labeled with BrdU 24 hours before measurement. BrdU incorporation was measured using the DELFIA Cell Proliferation Kit (PerkinElmer Life Sciences, MA, USA) according to the manufacturer’s protocol. Fluorescence was measured with a fluorescence detector (Infinite^®^ 200 PRO, TECAN). Data are expressed as absolute values of fluorescence detection, after background subtraction.

### Binding of Annexin-V and propidium iodide incorporation

For the detection of apoptosis cells were plated in 6-well plates and treated for 24, 48 and 72 hours as described above. Living cells were stained with Annexin V-FITC and propidium iodide (PI), according to manufacturer’s instructions (Annexin V-FITC kit, Società Italiana Chimici, divisione scientifica S.r.l., Roma, Italy). Cells were analyzed using a flow cytometer (FACSCalibur, BD Bioscience, Italy) and separated according to their specific feature, namely: i) normal cells (annexin V negative and PI negative); ii) early apoptotic cells (annexin V positive and PI negative; iii), necrotic cells or late apoptotic cells (annexin V positive and PI positive). Analysis was performed using 488 nm excitation and 515 nm band pass filter for FITC detection and a filter > 600 nm for PI detection. Data have been analyzed utilizing Cell Quest Pro software (BD Biosciences, Italy).

### Protein analysis

Cells were plated in 25 cm^2^ flasks and treated for 12 and 24 hours for caspase measurements and for 3, 6 and 12 hours for NF-κB nuclear level quantification. For total protein extraction, cells were pelleted and then resuspended in lysis buffer (150 mM NaCl, 5 mM EDTA, 1 mM EGTA, 1% Tryton X-100, 10 mM Tris-HCl pH 7.4, protease inhibitor cocktails). Protein quantification was performed according to Lowry assay. The purification of nuclear proteins was performed as previously described [17]. 30 μg of total extracts and 10 μg of nuclear proteins were resolved using SDS/Mini-PROTEAN TGX Precast gels of 4–20% range (Bio-Rad Laboratories, CA, USA). Proteins were then electro-transferred onto a polyvinylidene fluoride (PVDF) 0.2 μm membrane, using the Trans-Blot^®^ TurboTM Blotting System (Bio-Rad Laboratories, CA, USA). The cytosolic and particulate fractions were fractioned as previously described [18]. Proteins were incubated with the indicated antibodies: NF-κB p65, P-Bcl-2 (Ser 70), lamin B, Na+/K+ ATPase and GLUT-1 (sc-109, sc-21864, sc-365214, sc-21712, sc-1605 Santa Cruz Biotechnology Inc, Germany), Cleaved Caspase-3 (Asp175), Caspase-3, Caspase-7 and Caspase-9, (9915, Apoptosis antibody Sampler Kit, Cell Signaling Technology, Inc., MA, USA), Cleaved Caspase-8 (Asp391) and Caspase-8 (18C8 and 1C12, Cell signaling Technology, Inc., MA, USA); anti-α-tubulin (ab15246, Abcam, Cambridge, England). Proteins were detected with horseradish-peroxidase-conjugated secondary antibodies (GE Healthcare, Italy) and enhanced chemiluminescence reagent (ECL) (GE Healthcare Italy), followed by analysis and quantification of the chemiluminescence with the CCD camera detection systemLas4000 Image Quant (GE Healthcare, Italy).

### Caspase-3 enzymatic assay

Cells were plated in 6-well plates and treated for 12, 24 and 36 hours, as described above. The activity of caspase-3 was assessed using the Caspase-3/CPP32 Fluorimetric Assay Kit (BioVision Inc., CA, USA) according to the manufacturer’s instruction.

### NF-κB pathway array

Cells were plated in 6-well plates and treated for 12 hours as described above. At the end of the incubation, RNA was extracted and isolated using the RNAeasy^®^ Plus Mini kit (Qiagen, Netherlands) according to the manufacturer’s protocol. Concentration, purity and integrity were assessed using the Agilent 2100 Bioanalyzer (Agilent Technologies, CA, USA), before performing gene expression analysis. A total of 1 μg of isolated RNA samples was reverse-transcribed into cDNA using the iScript^™^ Reverse Transcription Supermix for qPCR (Bio-Rad Laboratories, CA, USA). The differential expression of 90 genes related to NF-κB pathway was evaluated using a Human NF-κB Signaling 96 StellARray^™^ qPCR array (Lonza Group, Switzerland). The quantification of gene expression was determined by real time PCR with the 7500 Fast Real-Time PCR System (Applied Biosystem, CA, USA) using the iTaqTM Fast SYBR^®^ Green Supermix with ROX (Bio-Rad Laboratories, CA, USA). For GLUT-1 expression the following primers were used: Fwd: 5′-AAGTCCTTTGAGATGCTGATCCT- 3′; Rev 5′-AAGATGGCCACGATGCTCAGATA-3′. Data were collected using the 7500 Software v2.0.5 and given as threshold cycle (Ct). Ct values for each target and reference genes (Hs18s) were obtained and their difference calculated (ΔCt). The comparative calculation, ΔΔC_t_, was used to find the difference of the expression level between control and treated samples. Data are expressed as the median of log2 of Fold Change (FC) respect to control.

### Clustering and network analysis

Differentially expressed genes were clusterized with the Ward method utilizing the Euclidean distance metric. The best number of clusters was identified by estimating the silhouette score for each possible cut of the cluster tree and selecting the cut that produces the best silhouette. Reactome pathways enriched by modulated genes were determined utilizing the Reactome web tool and selecting pathways that contain at least 10 modulated genes enriched with a False Discovery Rate (FDR) < 0.05. The network of interacting genes was built with the Cytoscape software (http://www.ncbi.nlm.nih.gov/pubmed/17947979) mining interactions annotated in the Pathway Commons database [19].

### Statistical analysis

Data in figures and tables are expressed as mean ± SEM, unless otherwise indicated. Statistical evaluation of the data was performed by using one way ANOVA (according to Fisher *post hoc* test). A P value <0.05 was considered statistically significant.

## Results

### CA decreases glucose uptake without affecting GLUT1 mRNA expression and translocation to plasma membrane

**Fig 1:**
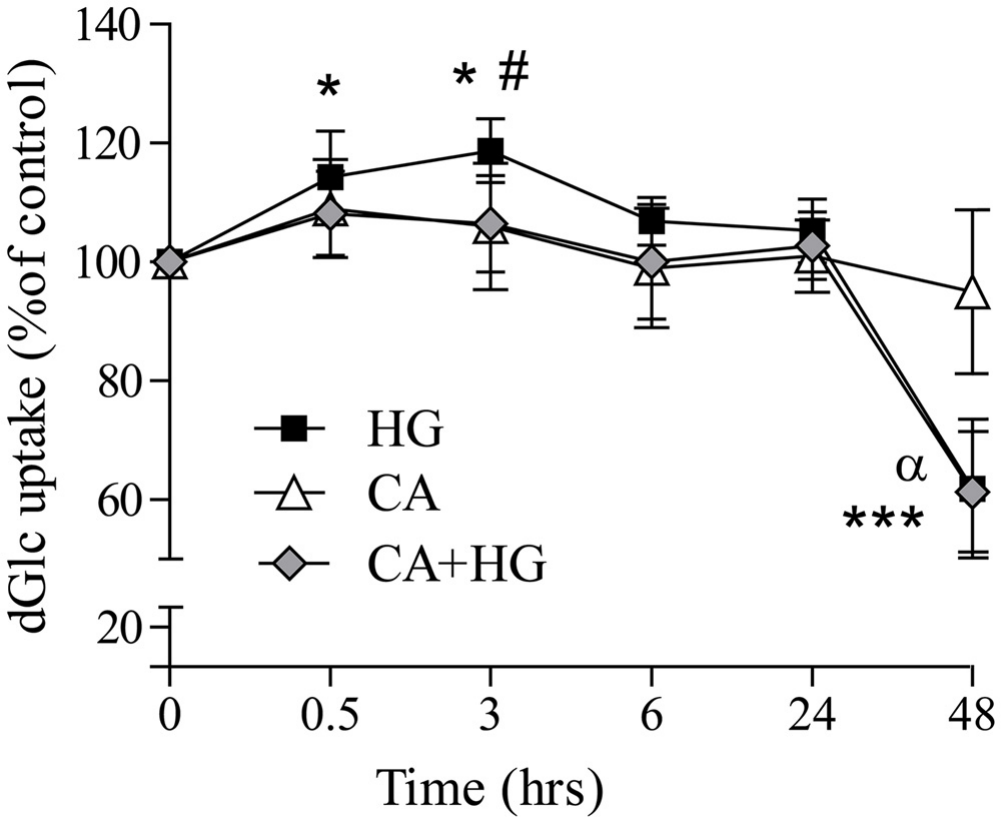
Effect of CA on glucose uptake. Sub-confluent cells were incubated in control, CA, HG or CA+HG conditions for 30 min, 3, 6, 24 and 48 hours. At the end of the incubation, cells were treated with 100 μM 2-dGlu and 0.1 μCi/ml 2-deoxy-D- [C14]glucose for 5 minutes. At the end of the incubation, radioactivity in cell lysate was detected by scintillation counting. The rate of glucose was expressed in pmol dGlu/min/μg protein and shown in the figure as % of control at each time point. Values are the mean ± SD from at least 3 independent experiments. *p<0.05 HG *vs* control; ***p<0.0001 *vs* control; #p<0.05 AC+HG *vs* HG; α p<0.001 versus CA.

In order to evaluate if CA could affect glucose transportation, we assessed the rate of 2-dGlu uptake as described in materials and methods. Fig 1 shows that after 30 minutes of HG incubation, the uptake of 2-dGlu was significantly increased respect to control, with a rate peak at 3 hours of treatment (20% of increase compared to control). CA significantly inhibited the increase of glucose entry induced by HG at 3 hours of incubation. At 48 hours of incubation, both HG and CA+ HG treatments were associated to a significant 40% decrease of glucose uptake with respect to control and CA treated cells. CA incubation alone was not associated to a significantly different rate of glucose entrance respect to control.

The increase of glucose uptake could be elicited either by a modulation of the expression of the glucose transporter GLUT-1 and/or by the regulation of its translocation to plasma membrane. However, no changes were observed neither in GLUT1 mRNA levels, analyzed at several time points, nor on the protein membrane localization analyzed at 3 hours of HG incubation by western blotting (S2 Fig).

### CA prevents the HG-induced increase of intercellular permeability and actin rearrangement

**Fig 2:**
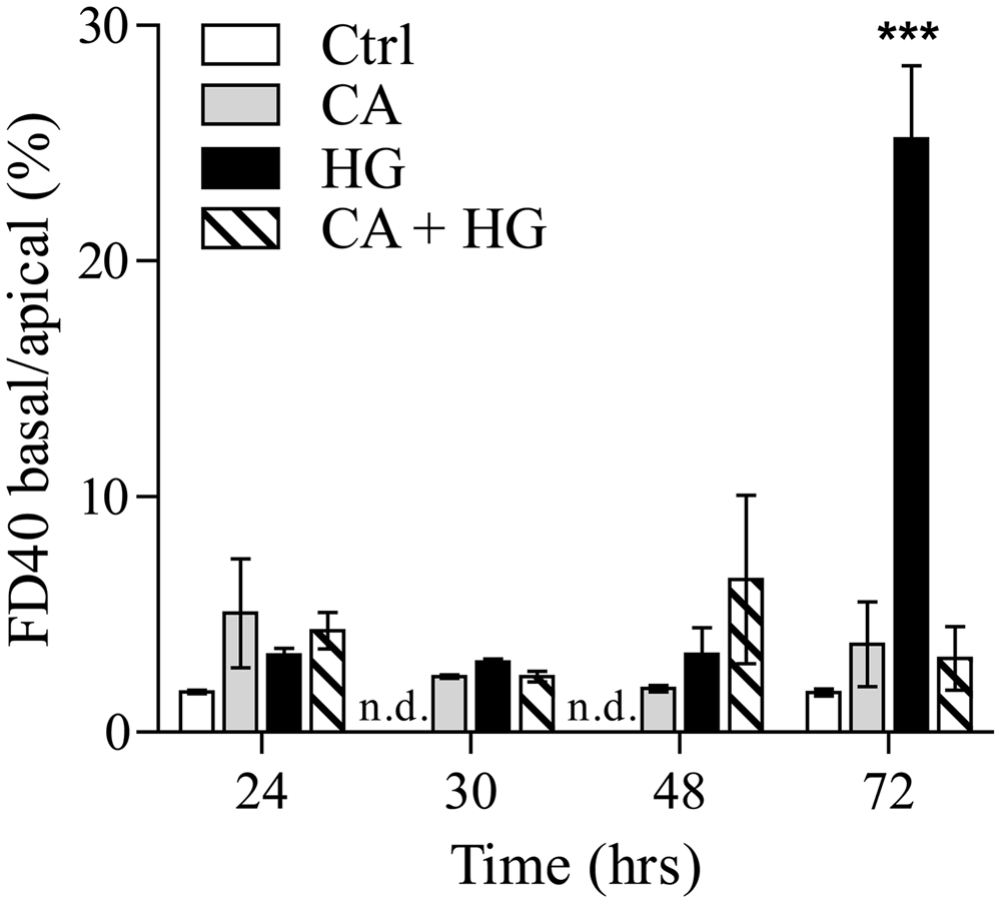
CA protects HG-dependent loss of endothelial barrier integrity. Cells were seeded onto PET Transwell filters and incubated in control, CA, HG and CA+ HG conditions for 24, 30, 48 and 72 hours. FITC-FD40 was added to a final concentration of 500 μg/ml in the apical medium. The fluorescence was measured both in basal and apical medium. Data are presented as percentage of the ratios of FD40 basal *vs* apical fluorescence. ***p<0.0001 *vs* control; n.d. = not detected. Values are the mean ± SEM from at least 3 independent experiments.

In order to evaluate the effect of HG on cell-cell contact and barrier integrity, the paracellular diffusion of dextran (40 kDa polymer) was monitored using a fluorescence detector (FITC). At 72 hours of HG incubation, we observed a significant increase of FD40 transit from the apical to the basal medium, indicating the irreversible loss of the barrier integrity because of the generation of membrane perforations. When CA was included in the incubation medium, no changes of the paracellular permeability were observed up to 72 hours of HG treatment (Fig 2).

**Fig 3:**
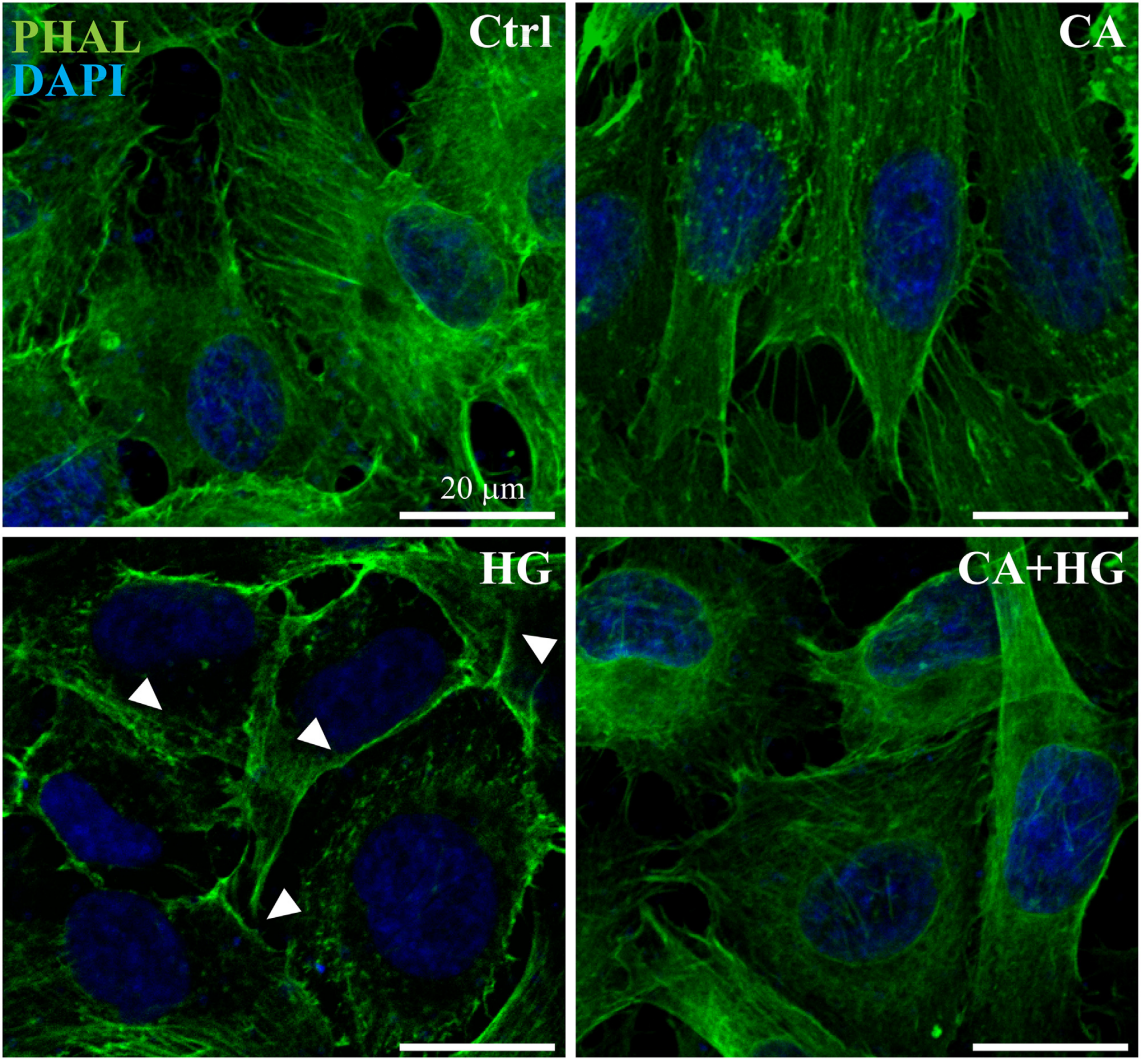
CA reduces HG-dependent actin reassessment. Cells were seeded onto PET Transwell filters and incubated in control, HG or CA+HG conditions for 48 hours. Cells were fixed in 2% PFA and permeabilized with 0.1% Triton X-100. F-actin filaments and nuclei were stained with FITC-conjugated phalloidin (green) and DAPI (blue), respectively. White arrows indicate peripheral F-actin. The image reports one out of at least 3 independent experiments.

Immunofluorescence staining demonstrated that after 48 hours of incubation, HG induced changes to the F-actin cytoskeleton, considered one of the early step of barrier disruption. In fact, in normal conditions, confluent Ea.hy926 cells have a polygonal shape and a typical polymerized actin distribution (F-actin), visible as marked cytoskeletal filaments (Fig 3). At 48 hours of HG treatment, changes in F-actin distribution were evident, resulting in a loss of cytoskeleton organization and in a shift of actin stress fibers to the cell border, eventually forming a distinct ring. CA alone did not affect actin reassessment while totally countered the effect of HG on actin filaments.

### CA counters the decrease of endothelial cell growth and reduces apoptosis induced by HG

**Fig 4:**
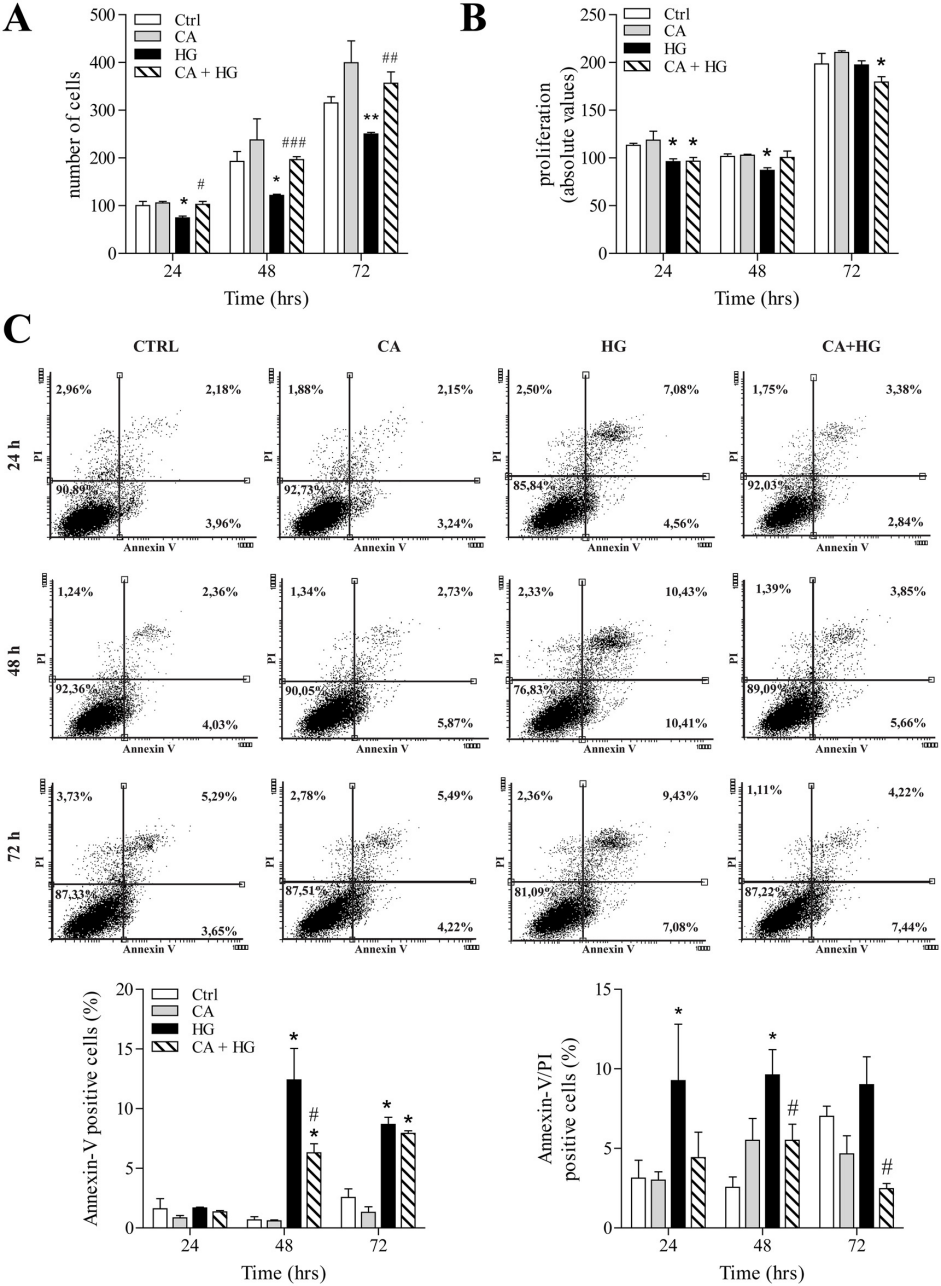
Effect of CA on cell proliferation and apoptosis. Cells were treated for 24, 48 and 72 hours as indicated in the figures. Panels show: A) cell count expressed as number of cells per well; B) Proliferation analysis by BrdU incorporation; C) Representative dot plots of cell distribution after double labeling with Annexin-V and PI. Data are expressed as percentage of induction of early (Annexin V positive cells) and late stages of apoptosis (Annexin-V/PI double positive cells). *p<0.05 HG *vs* control; **p<0.001 *vs* control; # p<0.05 CA+HG *vs* HG. ## p< 0.001 and ### p< 0.0001 CA+HG *vs* HG. Values are the mean ± SEM from at least 3 independent experiments.

In agreement with previous studies HG treatment was associated with a reduction of the number of cells over the time of incubation, with respect to control treatment (Fig 4A). Also in this case, CA totally countered the effect of HG, being associated with a complete preservation of cell number.

To evaluate if the HG-dependent decrease of endothelial cell number was due to a reduced proliferation rate or to increased apoptosis, we utilized BrdU incorporation and the cytofluorimetric assay of FITC-Annexin-V and PI. Our observations indicate that HG is associated with a decrease of cell proliferation at 24 and 48 hours of treatment and that co-treatment with CA abolished this effect only at 48 hours (Fig 4B). However, at 48 and 72 hours, the most evident effect of HG was the induction of phosphatidylserine (PS) exposure at the level of the outer membrane (Annexin V positive/PI negative cells) and the induction of late apoptosis/necrosis (Annexin V positive/ PI positive cells) (Fig 4C). In CA+HG treated cells the number of Annexin V positive cells significantly decreased with respect to HG at 48 hours, but it was still significantly higher in comparison to control cells. Anyway, CA induced a significant decrease of late apoptosis/necrosis phase in comparison to HG at 48 and 72 hours of HG co-incubation. In normoglycemic conditions, CA did not induce any significant change in cell number, proliferation or apoptosis rate, with respect to control cells.

### CA effects on caspase activation

**Fig 5:**
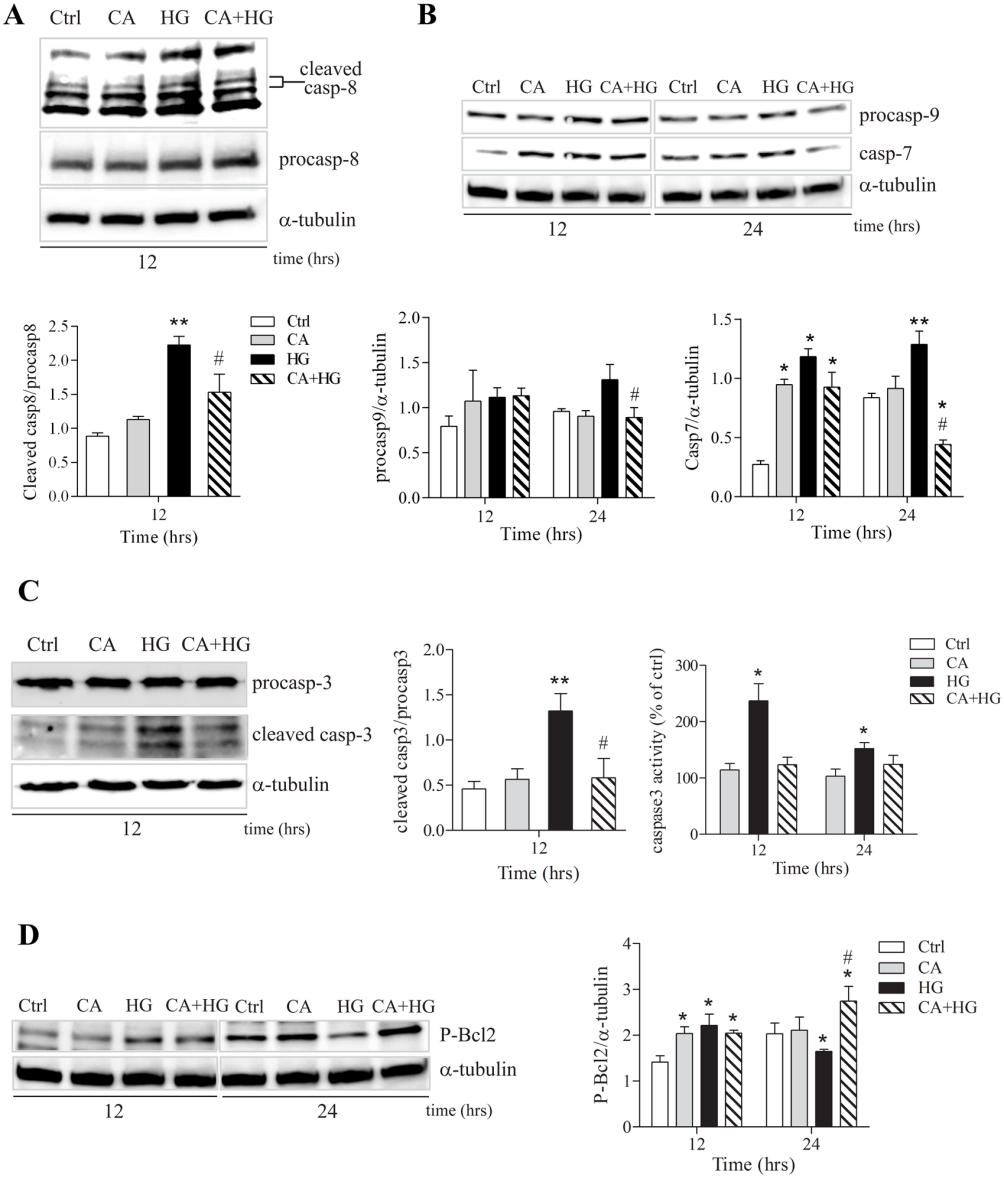
Analysis of activated caspases and anti-apoptotic Bcl-2. A) A representative Western blot of procaspase and cleaved caspase-8 after 12 hours of treatments. B) A representative Western blot of caspase-7 and procaspase-9 after 12 and 24 hours of treatments. C) A representative Western blot of procaspase and cleaved caspase-3 after 12 hours of treatments. Caspase 3 activity was measured by detection of fluorescence of the caspase substrate after 12, 24 and 36 hours of treatments. Data are expressed as relative AFC (7-amino-4-trifluoromethylcpumarin) fluorescence normalized for total protein content in each sample. D) Effect of CA on phosphorylated Bcl-2 protein levels at 12 and 24 hours of treatments. Images report one representative experiment out of at least 3 independent experiments.*p<0.05 *vs* control; **p<0.001 *vs* control; # p<0.05 CA+HG *vs* HG.

In order to provide a more accurate description of the effects of CA on HG induced apoptotic response, we assessed the expression levels of different pro-apoptotic and anti-apoptotic proteins. Shorter times of incubation were used in order to identify the upstream pathways associated to the functional assays performed at longer incubation times. observed endothelial dysfunction. Fig 5A and 5B show that CA significantly affected the activation of the initiator caspase-8 at 12 hours and pro-caspase-9 at 24 hours of HG incubation. On the other hand, irrespective to treatment, caspase-7 levels were significantly higher in comparison to baseline, at 12 hours of incubation After 24 hours, CA treatment determined a normalization of caspase-7 levels that were comparable to the ones detected under normoglycemic conditions. Remarkably, under hyperglycemic conditions, CA treatment was able to completely rescue the HG-induced caspase-7 activation, showing a significant reduction also compared with control cells (Fig 5B). Caspase-3 also plays a pivotal role in the apoptotic process and is usually the most important caspase, the activation of which is used as marker of programmed cell death. Thus, we assessed the activity and the protein levels of the cleaved form of caspase-3 by immunoblotting assay. Our data show that caspase-3 cleavage induced by HG was significantly inhibited by CA at 12 hour of incubation (Fig 5C). This observation was corroborated by the measurement of caspase-3 activity. In fact, in presence of CA, the activity of caspase-3 in HG treated cells was comparable to control cells up to 24 hours (Fig 5C).

Within the execution of an apoptotic response, Bcl-2 exerts its role as key regulator of apoptosis through a proteolytic process and phosphorylation (Ser70) [20]. For this reason, we analyzed the levels of the functional form of Bcl-2 (Ser70 phosphorylated form) at 12 and 24 hours of HG treatment. Irrespective to the treatment, at 12 hours the level of P-Bcl-2 was higher respect to baseline. However, at 24 hours from the administration of HG, P-Bcl-2 was significantly reduced compared to control cells, and the co-treatment with CA was associated with higher P-Bcl-2 levels, in comparison both with control and HG cells. Finally, P-Bcl-2 levels in the presence of CA alone were comparable to control cells (Fig 5D).

### The modulation of NF-κB activation by CA is associated to the inhibition of pro-apoptotic response induced by HG

**Fig 6:**
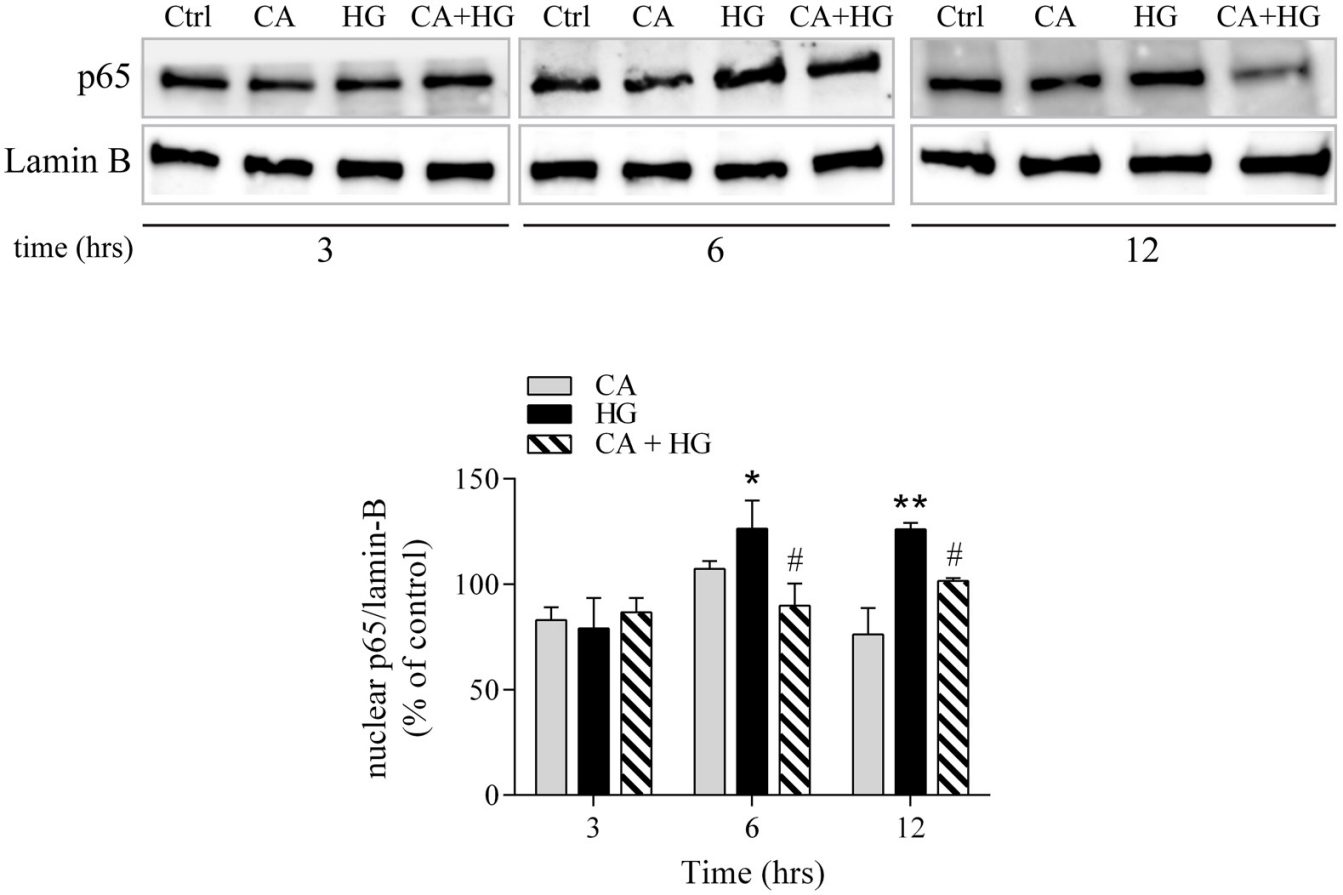
p65 nuclear localization. Cells were incubated with CA, HG or CA+HG for 3, 6 and 12 hours *p<0.05 *vs* control; **p<0.001 *vs* control; #p<0.05 CA+HG *vs* HG. The image reports one out of at least 3 independent experiments.

NF-κB is a well-known transcription factor that, upon different stimuli and in a cell-dependent manner, induces the transcription of several genes involved in proliferative, inflammatory or apoptotic pathways. Notably, hyperglycemia results in a pro-inflammatory and pro-apoptotic NF-κB activity in endothelial cells. In order to investigate if the molecular effects of CA were related to HG-dependent activation of NF-κB pathways, we measured the nuclear levels of the p65 subunit of NF-κB, at 3, 6 and 12 hours of incubation with HG. At 6 and 12 hours of HG incubation, nuclear levels of p65 were significantly higher than at baseline. CA does not have any significant effect on nuclear p65 NF-κB levels respect to control. Remarkably, we observed that co-treatment with CA significantly countered the activation of NF-κB, in particular at 12 hours of HG treatment (Fig 6).

**Fig 7:**
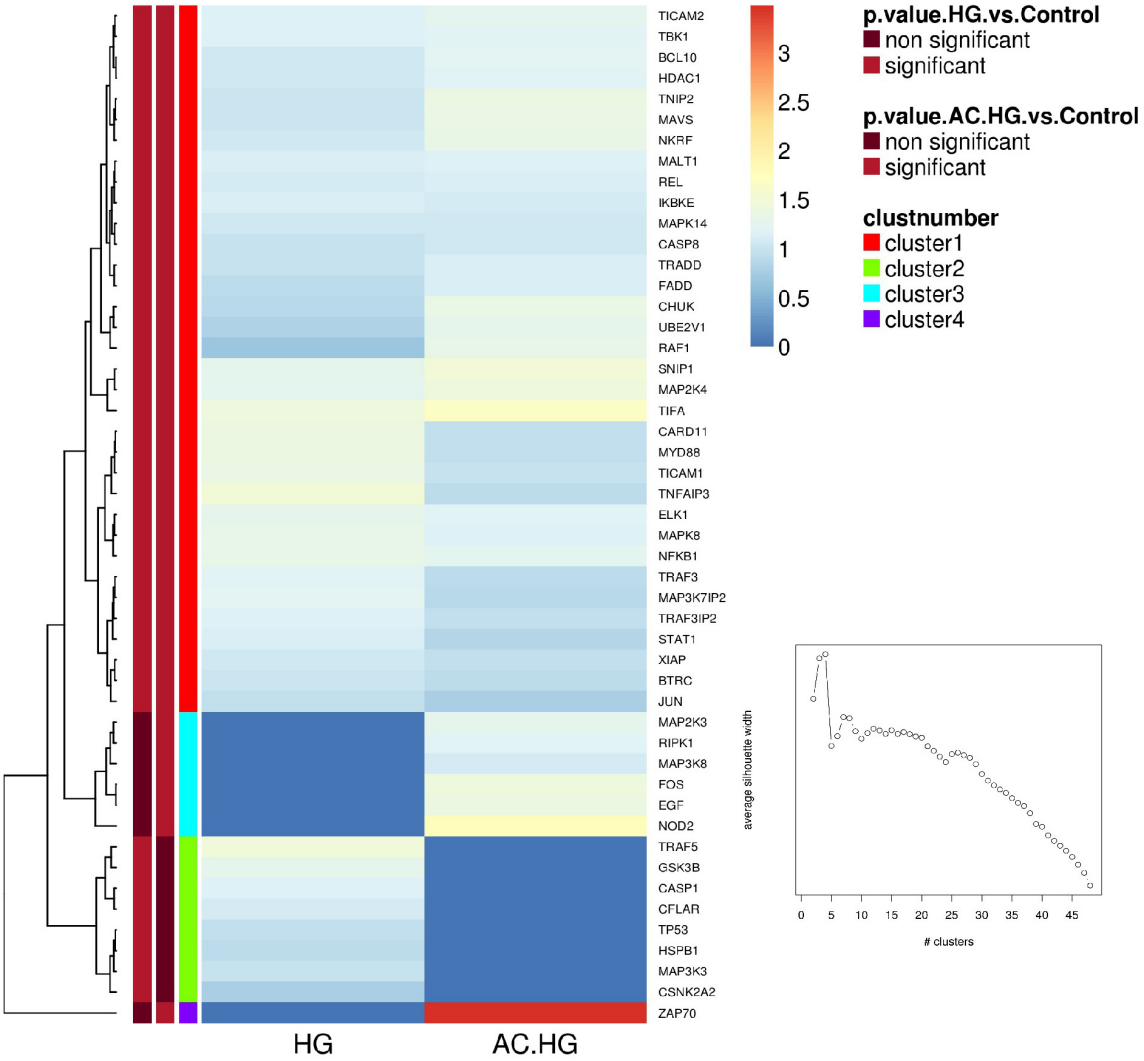
Heat map generated from StellARray^™^ qPCR data. The heatmap reflects the differently regulated gene expression values in CA+HG and HG (12 hours of incubation) respect to control. Color scheme goes from red for upregulated genes to blue for down-regulated genes. Data are expressed as the median of log2 of Fold Change (FC).

**Fig 8:**
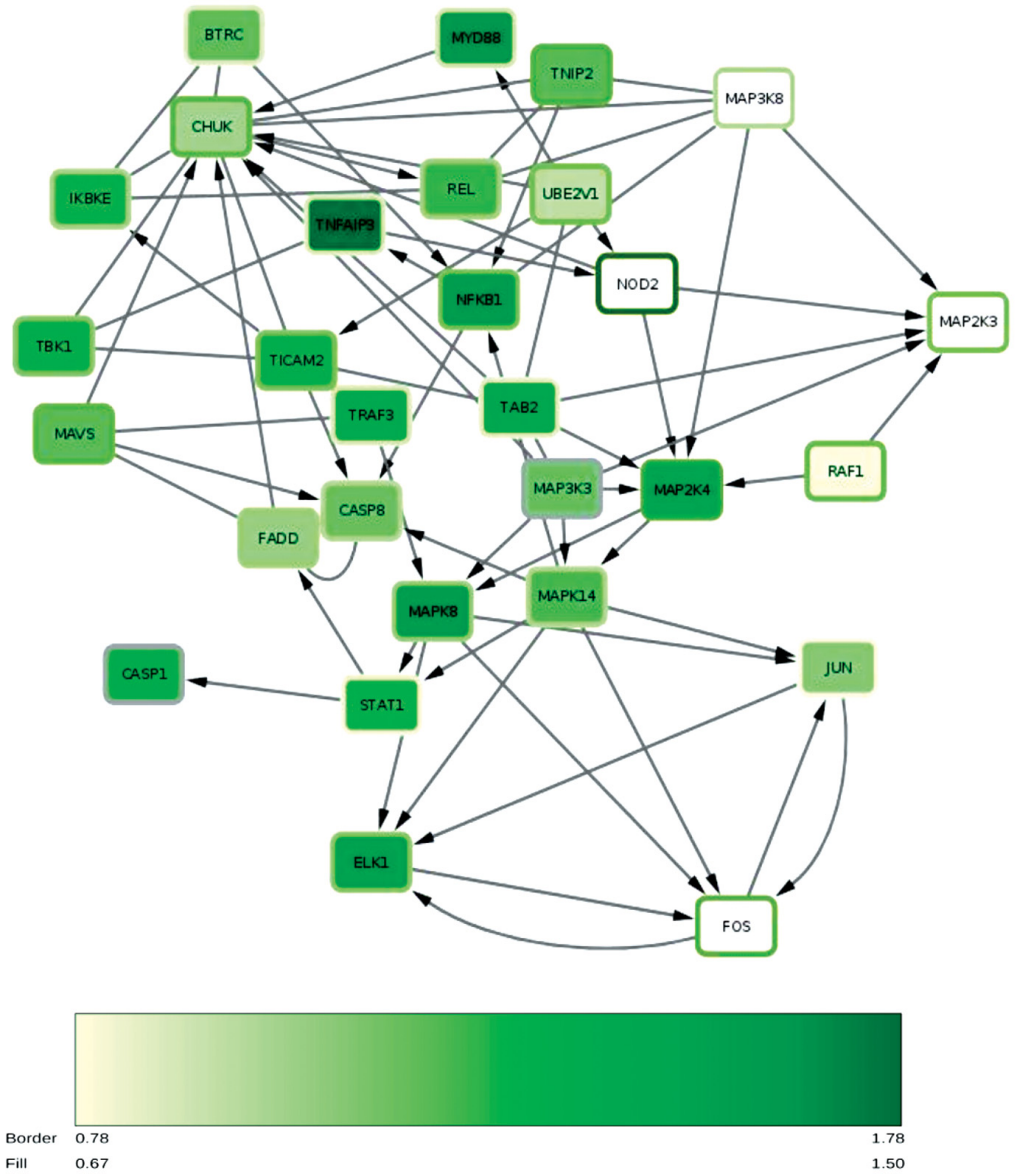
Mapping of the significant modulated genes within Reactome pathways database tool. The color of the nodes represents the FC expression associates to HG, the color of the frame of the nodes corresponds to the FC expression associated to AC+HG. The color intensity of the nodes is associated to the FC rate as indicated in the figure.

A Human NF-κB Signaling 96 StellARray^™^ qPCR arrays was utilized to study the molecular pathways involved in the modulation of NF-κB activity by CA and to confirm its role in endothelial dysfunction. This methodological tool allows the evaluation of the differential expression of about 90 genes related to NF-κB pathway. Ea.hy926 cells were treated for 12 hours in control, HG or CA+HG conditions. (Numerical values of FC and P values are presented in S1 Table). Fig 7 shows a “heat map representation” of the FC expression of the genes differentially modulated by the two treatments, HG and CA+HG respect to control. Out of 90 genes analyzed, 43 were significantly up-regulated at 12h of HG treatment respect to baseline. In the presence of CA, HG treatment was associated to a differential expression of a total of 44 genes, 36 shared with the HG treatment. Noteworthy, 9 genes were significantly regulated in a different manner by CA+HG compared to HG (S1 Table). Namely in CA+HG cells, CARD11, MAP3K3 and TP53 were significantly down-regulated, while CHUK, CSNK2A2, MAP3K8, NKRF, RAF1 and ZAP70 were significantly up-regulated, in comparison to HG cells. Within this set of genes differentially regulated by HG in the presence of CA, NKRF encodes for the NF-κB -Repressing Factor that interacts with specific Negative Regulatory Elements (NREs) and mediates the transcriptional repression of a number of NF-κB -responsive genes [21]. RAF-1, one of the three RAF (Rapidly Accelerated Fibrosarcoma) kinases that are key regulators of the mitogen-activated protein kinase enzyme (MEK—MAPK/ERK) cascade, is involved in interleukins signaling; Raf proteins are proto-oncogenes, and their activation is primarily related to the regulation of growth, proliferation, differentiation or apoptosis [22]; the Conserved Helix-loop-helix Ubiquitous Kinase (CHUK) is another gene that we found to be significantly up-regulated by CA in hyperglycemic condition; CHUK encodes for the subunit IKKα, a part of the IκB kinase complex that plays an important role in regulating NF-κB transcription factor activity [23]; CARD 11 is a component of the CARD11-BCL10-MALT1 signalosome complex that is linked to NF-κB activation [24]. Finally, it is also important to mention TP53 that is involved in cell cycle regulation acting as a tumor suppressor gene, but it has also been reported to contribute to LPS-induced apoptosis in endothelial cells [25]. To better understand the effect of HG on gene expression, the differentially expressed genes were mapped using the REACTOME pathway database tool and the following regulated pathways were identified: MYD88 dependent and independent pathway (REACT_25222 and REACT_6809, respectively), the toll like receptor (TLR) cascades (REACT_ 6966) and RIG-I/MDA5 mediated induction of IFN-alpha/beta pathways (REACT_25222). Fig 8 shows the network of the genes modulated by HG and CA+ HG (and the related intensity of expression respect to control) involved in those pathways.

## Discussion

Several epidemiological studies have associated moderate and long term consumption of coffee with a reduced risk of developing T2D [12, 26]. The available literature indicates that important differences exist between the potentially negative effects of acute coffee consumption, mainly due to the cardio-dynamic effects of caffeine [27], in comparison with a life-long regular though moderate consumption of coffee. However, it is important to remark that caffeine represents only the 2% of the total coffee’s chemical profile and the coffee-mediated beneficial effects have been associated to its content of phenolic compounds [28] and in particular to CA, suggesting this molecule as one of the most bio-active components of coffee [29, 30].

The aim of this study was to investigate the effect of physiological achievable concentration of CA (10 nM) on endothelial dysfunctions induced by HG and to identify the molecular mechanisms elicited by CA and involved in cellular response. For this purpose, we have utilized Ea.hy 926 cells, a line obtained by the fusion of the human lung adenocarcinoma cells A549, with genuine HUVECs. This line is widely utilized as an expedient experimental model for the vascular cells as it maintains the same morphology and response characteristics very similar to those displayed by primary endothelial cells [15, 31]. We have exposed cells to a medium containing 25 mM or 5 mM glucose. Morphology was taken under observation together with several molecular markers of cell viability and function.

Cellular glucose transport is a highly regulated process mediated by specific transporters (GLUTs). GLUT1 is an insulin independent transporter and therefore is responsible for basal glucose uptake and also, the most abundant transporter isoform in different cell types, including endothelial cells. In agreement with other studies, our data show that the incubation of the cells with HG rapidly induces an increase of the rate of glucose entrance [32] that reverts back and below to the background levels after 48 hours [33–35]. Very high concentrations of CA (1 mM) have been shown to modulate cellular glucose uptake either positively [36] or negatively [37], according to experimental design and cellular types. Notably, in the present study, 10 nM CA, a concentration easily achievable in plasma following a phenolic rich meal or after a normal coffee consumption, significantly reduced glucose entrance at 3 hours of incubation in HG. The observed changes of glucose uptake, both in HG or CA+HG were not associated with any changes of GLUT-1 mRNA expression or protein membrane localization. These observations are in agreement with previous studies addressing the effect of HG [32], but discordant with others [38, 39]. The observed presence of a reduced glucose uptake, associated to a lack of effects on GLUT1 expression and membrane translocation can be explained by hypothesizing either a direct “physical” interaction of this molecule with GLUT-1 protein, and therefore with the transport activity, or the induction of the recruitment of other glucose transporters. Further investigations are warranted to confirm these hypotheses.

Starting from the evidence of a reduced glucose uptake, in order to further understand the molecular basis of the effects of CA in HG-induced endothelial dysfunction, we have studied the changes of cell morphology and the effects on viability [40, 41]. One of the functions of the endothelium is to act as a selective barrier between plasma and the interstitial space. Thus, one of the most evident effects of endothelial dysfunction is the loss of the selective permeability, which in turn is associated with an uncontrolled flow of molecules throughout the interstitial space [4]. Our observations indicated that the presence of CA totally counters the HG-induced loss of barrier integrity and the increase of non-selective permeability. Immunofluorescence experiments corroborated this observation indicating that the protective effect of CA on cell permeability is due, at least in part, to its ability to sustain and counteract the organization of actin into contractile fibers under HG conditions [4, 42]. Under prolonged HG conditions, modifications of cell morphology and integrity are accompanied by the initiation of a program leading to apoptosis. Previous studies reported that increased cell death, rather than reduced proliferation, is one of the major outcomes associated to HG [43, 44]. Our data indicates that, in HG conditions, the presence of CA reduces both the exposure of PS to the outer layer of the membrane up to 48 hours of incubation and the number of Annexin V positive cells and Annexin V/PI double positive cells, which are markers of early and later stages of apoptosis, respectively. The capacity of CA to counter HG induced apoptosis is further confirmed by the observation that caspase-3 cleavage and activity are also reduced in the presence of CA. Caspase-3, an executor caspase, is an important marker of programmed cell death since it tags the entry of cells into the apoptotic signaling pathway. Its activation is related to both caspase-8 and caspase-9, involved in the extrinsic and intrinsic pathways, respectively and with the exposure of PS on the outer membrane [45]. However, the increase of the expression of the executor caspase-7 after 12 hours of incubation and the increase of PS exposure on the outer membrane with respect to the baseline indicated that the presence of CA was not able to totally counter the activation of the apoptotic pathways induced by HG. Taken together, these results suggest that, although the rate of glucose entrance measured in CA+HG is statistically comparable to baseline, the rate of glucose uptake is sufficient to activate a different cellular response respect to control.

Accordingly to the report that unphosphorylated Bcl-2 may not be sufficient for the protection from apoptosis [20], HG treatment was reported to not affect total Bcl-2 protein expression [41]. We thus analyzed the amount of the functional phosphorylated form of the protein and found an increased phosphorylation at 12 hours of incubation, irrespective to the treatments. Later, the activation of the protein was maintained at high levels only in CA+HG treatment, supporting the cellular anti-apoptotic response to HG. On the other hand, the significant decrease of phosphorylated Bcl-2 in HG endures the pro-apoptotic pathway. Bcl-2 is known to protect cells from apoptosis by activating downstream the release of mitochondrial cytochrome-c, thereby preventing caspase-3 dependent proteolytic cascade [46]. Since a cross-talk exists between Bcl-2 intrinsic and extrinsic pathways [47], the activation of Bcl-2 affects the increase of expression and activity of caspase-3 associated to HG treatment. It is interesting to note that elevated, non-physiological concentrations of CA (1 or 10 mM) have been shown to induce apoptosis by inhibiting Bcl-2 activity [48]. As mentioned above, the study described herein was conducted considering CA at concentrations up to 100,000 fold lower than those utilized in previous studies. It is therefore plausible that largely higher doses can be associated to significantly different, possibly opposite, effects. It is worth nothing that the incubation of endothelial cells with CA in normoglycemic condition was not associated to any significant changes of glucose uptake or permeability, but at the molecular level we observed, after 12 hours of incubation, an increase of caspase-7 and phosphorylated Bcl-2 protein expression, suggesting that 10 nM CA induces a cellular response that is buffered within a homeostatic physiological equilibrium. In fact, the treatment with CA did not induce any detectable decrease of cell number or increase of PS exposure on the outer membrane.

Previous studies reported an endothelial-specific correlation between the induction of apoptosis by HG and the activation of the NF-κB pathway [44, 49]. Our data confirmed that HG is associated to a significant increase of NF-κB nuclear levels and demonstrated that CA quenches this response, as suggested by the decreased nuclear localization of p65.

Mapping of HG-differentially expressed genes, resulting from the StellARray^™^ qPCR array analysis, in the REACTOME database tool highlights the apoptotic and inflammatory pathways activated by HG that notably include the TLR and RIG-I/MDA5 signaling pathways. TLR signaling is triggered either *via* a MYD88 dependent or MYD88 independent pathways [50]. MYD88 mediates both NF-κB activation or apoptosis, *via* a pathway involving FADD and CASP8 [50, 51]. Activated CASP-8 cleaves and activates CASP-3, which, in turn, executes the apoptotic program. Likewise, the MYD88 independent pathway induces apoptosis by triggering the signal activation of both extrinsic and intrinsic pathway [52]. RIG-I/MDA5 pathway, similarly to MYD88, triggers the production of IFNs by activating different transcription factors, including NF-κB and the interferon regulatory factor-3 (IRF3). This latter is also associated to the activation of caspase cascade [53, 54]. The presence of CA modulated the expression of several genes involved in the signaling response associated to HG, as described in the result section. Since NF-κB activation has been shown to lead to apoptosis in HG treated cells, modulation of those signaling pathway related to NF-κB and the decrease of the protein nuclear levels respect to HG can be the responsible of the observed reduction of the pro-apoptotic program associated with CA.

In conclusion, our data demonstrate that low, physiological concentrations of CA, significantly reduce the potentially detrimental effects elicited by HG on cultured endothelial cells. The CA-dependent decrease of the glucose flux inside the cell can be considered a pivotal mechanism of cellular protection toward high concentration of glucose. Accordingly, a decreased metabolic stress associated to the presence of CA appears able to modify the cell response to HG, allowing the activation of survival mechanisms, through increased phosphorylated Bcl-2 levels, modulation of NF-κB signal transduction and reduction of p65 subunit nuclear translocation.

## Supporting Information

S1 Fig Effect of 5, 10 and 100 nM of CA on cell viability. (TIF)

S2 Fig GLUT1 mRNA expression and protein membrane localization. (TIF)

S1 Table Numerical values of the expression analysis of the differentially modulated genes. (DOCX)
