# Supplementary figures and images for: Nanomolar Caffeic Acid Decreases Glucose Uptake and the Effects of High Glucose in Endothelial Cells
Source: PLoS One. 2015 Nov 6;10(11):e0142421. doi: 10.1371/journal.pone.0142421 (PMC4636304; doi:10.1371/journal.pone.0142421)

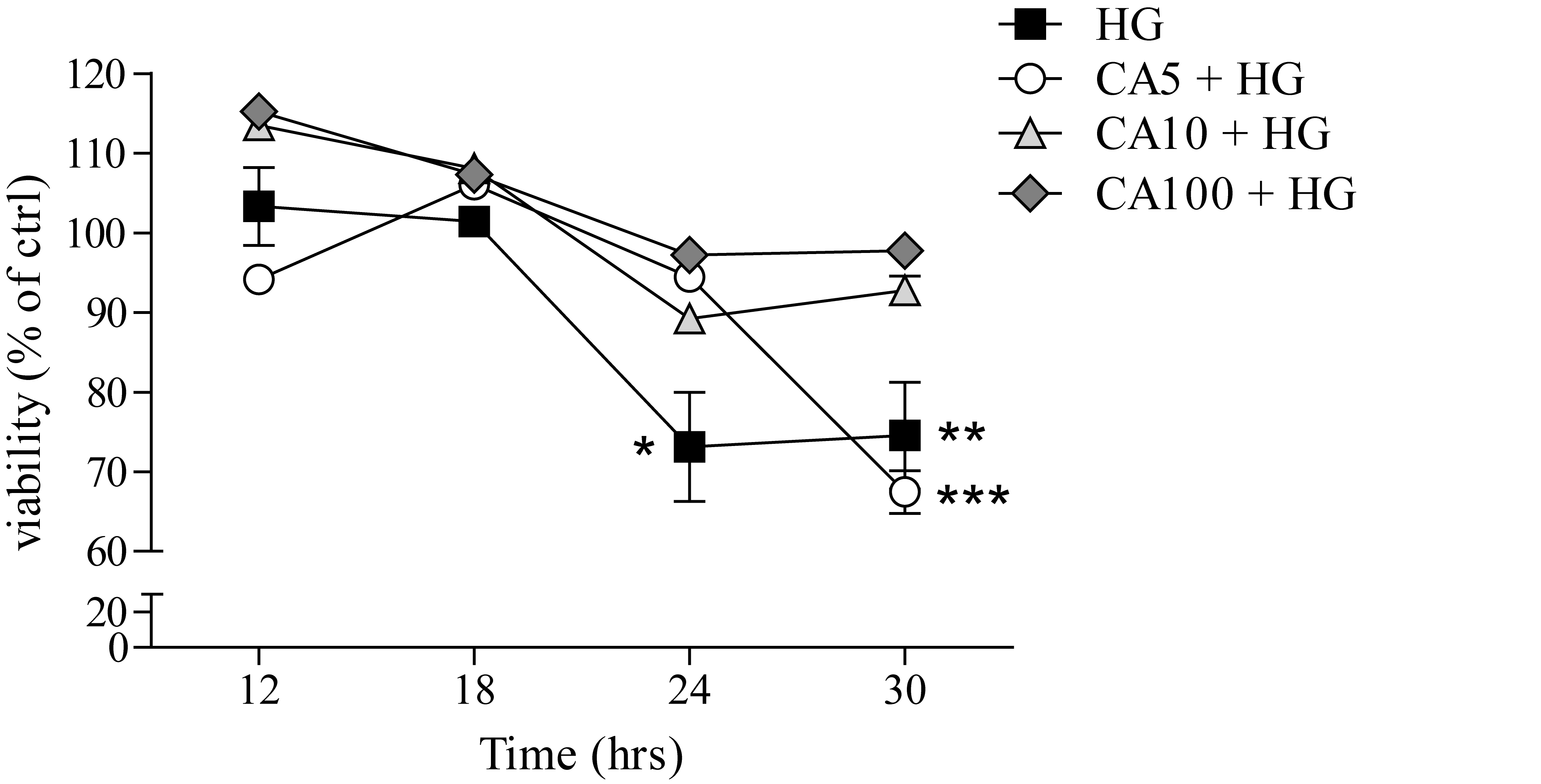

Supplement: S1 Fig — (TIF) [file pone.0142421.s001.tif]

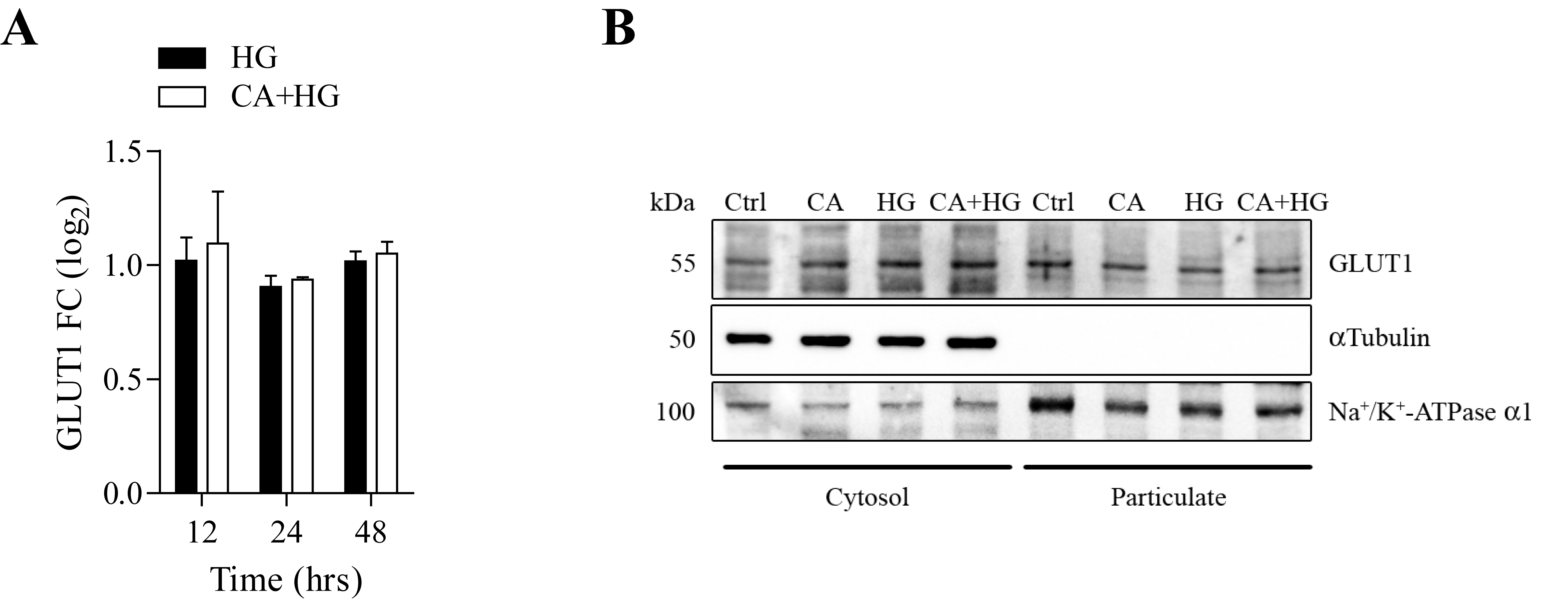

Supplement: S2 Fig — (TIF) [file pone.0142421.s002.tif]
