# Supplementary material for: Nanomolar Caffeic Acid Decreases Glucose Uptake and the Effects of High Glucose in Endothelial Cells
Source: PLoS One. 2015 Nov 6;10(11):e0142421. doi: 10.1371/journal.pone.0142421 (PMC4636304; doi:10.1371/journal.pone.0142421)
Supplement: S1 Table — (DOCX) [file pone.0142421.s003.docx]

|  |  | HG | P value | AC+HG | P value | P value |
| --- | --- | --- | --- | --- | --- | --- |
|  | GENE ID | (log2FC) | (HG vs C) | (log2 FC) | (AC+HG *vs* C) | (AC+HG *vs* HG) |
| BCL10 | *603517 | 1,053 | 0,003 | 1,214 | 0,005 | ns |
| BTRC | *603482 | 1,013 | 0,016 | 0,902 | 0,026 | ns |
| CARD11 | *607210 | 1,413 | 0,001 | 0,959 | 0,025 | 0,044 |
| CASP1 | *147678 | 1,169 | 0,03 | 0,717 | ns | ns |
| CASP8 | *601763 | 0,997 | 0,029 | 1,060 | 0,030 | ns |
| CFLAR | *603599 | 1,090 | 0,02 | 0,464 | ns | ns |
| CHUK | *600664 | 0,876 | 0,003 | 1,358 | 0,000 | 0,050 |
| CSNK2A2 | *115442 | 0,788 | 0,001 | 1,293 | <0,0001 | 0,010 |
| EGF | *131530 | 0,420 | ns | 1,408 | 0,042 | ns |
| EGR1 | *128990 | 1,058 | ns | 2,041 | 0,064 | ns |
| ELK1 | *311040 | 1,303 | 0,001 | 1,198 | 0,001 | ns |
| FADD | *602457 | 0,897 | 0,005 | 1,113 | 0,006 | ns |
| FOS | *164810 | 1,033 | ns | 1,442 | 0,050 | ns |
| GSK3B | *605004 | 1,291 | 0,047 | 0,807 | ns | ns |
| HDAC1 | *601241 | 1,056 | 0,003 | 1,200 | 0,002 | ns |
| HSPB1 | *602195 | 0,910 | 0,027 | 0,702 | ns | ns |
| IKBKE | *605048 | 1,114 | 0,016 | 1,086 | 0,020 | ns |
| JUN | *165160 | 0,937 | 0,004 | 0,787 | ns | ns |
| MALT1 | *604860 | 1,105 | 0,003 | 1,162 | 0,004 | ns |
| MAP2K3 | *602315 | 1,216 | ns | 1,294 | 0,038 | ns |
| MAP2K4 | *601335 | 1,274 | 0,004 | 1,437 | 0,002 | ns |
| MAP3K3 | *602539 | 0,999 | 0,002 | 0,406 | ns | 0,006 |
| MAP3K7IP2 | *605101 | 1,226 | 0,006 | 0,878 | 0,010 | ns |
| MAP3K8 | *191195 | -0,333 | ns | 1,089 | 0,040 | 0,018 |
| MAPK14 | *600289 | 1,051 | 0,012 | 1,062 | 0,036 | ns |
| MAPK8 | *601158 | 1,342 | 0,000 | 1,145 | 0,000 | ns |
| MAVS | *609676 | 1,021 | 0,005 | 1,353 | 0,006 | ns |
| MYD88 | *602170 | 1,384 | 0,005 | 0,942 | 0,009 | ns |
| NFKB1 | *164011 | 1,343 | 0,002 | 1,252 | 0,008 | ns |
| NKRF | *300440 | 1,066 | <0,0001 | 1,313 | <0,0001 | 0,012 |
| NOD2 | *****605956 | 0,419 | ns | 1,788 | 0,050 | ns |
| PP1R13L | *****607463 | 0,920 | 0,051 | 1,621 | 0,062 | ns |
| RAF1 | *164760 | 0,669 | 0,004 | 1,334 | 0,000 | 0,048 |
| REL | *164910 | 1,076 | 0,004 | 1,124 | 0,022 | ns |
| RIPK1 | *****603453 | 0,720 | ns | 1,195 | 0,015 | ns |
| SNIP1 | *608241 | 1,295 | 0,032 | 1,491 | 0,032 | ns |
| STAT1 | *600555 | 1,123 | 0,025 | 0,836 | 0,043 | ns |
| TBK1 | *604834 | 1,142 | 0,001 | 1,199 | 0,002 | ns |
| TICAM1 | *607601 | 1,370 | <0,001 | 0,998 | 0,000 | ns |
| TICAM2 | *608321 | 1,143 | 0,003 | 1,243 | 0,003 | ns |
| TIFA | *609028 | 1,445 | 0,001 | 1,686 | 0,000 | ns |
| TNFAIP3 | *191163 | 1,502 | 0,002 | 0,905 | 0,013 | ns |
| TNIP2 | *610669 | 1,029 | 0,007 | 1,382 | 0,003 | ns |
| TP53 | *191170 | 0,943 | 0,05 | 0,114 | ns | 0,050 |
| TRADD | *603500 | 0,974 | 0,013 | 1,123 | 0,021 | ns |
| TRAF3 | *601896 | 1,197 | 0,01 | 0,911 | 0,045 | ns |
| TRAF3IP2 | *607043 | 1,163 | 0,001 | 0,951 | 0,003 | ns |
| TRAF5 | *602356 | 1,459 | 0,022 | 0,704 | 0,119 | ns |
| UBE2V1 | *602995 | 0,815 | 0,023 | 1,285 | 0,015 | ns |
| XIAP | *300079 | 1,054 | 0,008 | 0,931 | 0,008 | ns |
| ZAP70 | *176947 | 0,420 | ns | 3,482 | 0,040 | 0,050 |
